# Energy balance-related factors associating with adolescent weight loss intent: evidence from the 2017 National Youth Risk Behavior Survey

**DOI:** 10.1186/s12889-019-7565-8

**Published:** 2019-09-02

**Authors:** Ryan D. Burns

**Affiliations:** Department of Health, Kinesiology, and Recreation, University of Utah, 1850 East 250 South Room 251, Salt Lake City, UT 84112 USA

**Keywords:** Diet, Energy balance, Physical activity, Weight loss, Survey

## Abstract

**Background:**

The purpose of this study was to examine specific energy balance-related behaviors (sedentary behaviors, physical activity, and dietary) associating with adolescent weight loss intent using data from the 2017 US Youth Risk Behavior Survey (YRBS).

**Methods:**

This was a cross-sectional study that employed a multi-stage cluster sampling procedure to obtain a representative sample of US adolescents. The target population consisted of public and private high schoolers from grades 9 through 12. The number of sampled adolescents was 18,324 with 14,765 of the 18,324 sampled students (Mean age = 15.9 (1.3) years) submitting questionnaires with usable data (81% response rate). The outcome was intent to lose weight with specific energy-balance related behaviors examined as predictor variables. A weighted logistic regression model was employed to examine the associations between sedentary behaviors, physical activity, and dietary-related variables with weight loss intent controlling for age, sex, BMI percentile, and race/ethnicity.

**Results:**

Variables associating with adolescent intent to lose weight included 3 or more hours of video game playing (OR = 1.15, 95%CI: 1.01–1.31, *p* = 0.028), achieving 60 min of physical activity daily (OR = 0.66, 95%CI: 0.59–0.73, *p* < 0.001), daily breakfast consumption (OR = 0.76, 95%CI: 0.67–0.87, *p* < 0.001) and weekly salad consumption (OR = 1.30, 95%CI: 1.12–1.52, *p* = 0.001).

**Conclusions:**

Meeting physical activity guidelines and regular breakfast consumption associated with lower odds of weight loss intent and video game playing and salad consumption associated with higher odds of weight loss intent in a representative sample of US adolescents. Therefore, there is a discordance between adolescent weight loss intent and the engagement in specific energy balance-related health behaviors, particularly physical activity.

## Introduction

Adolescent obesity is continuing to be a major public health problem, with a US obesity prevalence of 20.6%, adolescents are at risk for poor health [1, 2]. Many nations throughout the world have identified youth overweight and obesity as not only a health issue but also an economic issue because of the high health care costs accompanying excess body weight [3, 4]. Overweight and obesity are commonly defined as an excess of body fatness that compromises health and daily function [5]. Although not without limitations at the individual level, Body Mass Index (BMI), a ratio of weight (in kilograms) to height squared (in meters), commonly serves as a screening measure for overweight and obesity in both youth and adults [6]. Excess body weight is thought to be primarily a function of positive energy-balance where total energy (kilocalories) consumed is consistently higher than total energy expended; thus, energy balance-related health behaviors greatly influence overweight and obesity risk [7].

Numerous interventions have been derived to prevent overweight and obesity in the pediatric population [8]. It has been recommended that programs should focus on targeting three primary behaviors of energy balance: sedentary behaviors, physical activity, and diet [9]. It has been shown that multicomponent community-based interventions targeting change in multiple behaviors at various times throughout the day may be the most effective strategy to prevent overweight and obesity [10]. While many of these interventions focusing on overweight and obesity prevention has yielded effectiveness [11], treatment of overweight and obesity (i.e., weight loss) tends to be a more difficult outcome to improve. Indeed, it has been shown that initial observed weight loss during multidisciplinary obesity treatment trials tends to be unsustainable in morbidly obese adolescents [12].

Although there is potential for community-based multicomponent and multi-behavioral interventions for overweight and obesity treatment, it is unclear what specific energy balance health behaviors adolescents tend to engage in if they do have intent to lose weight. Meeting daily physical activity guidelines and reducing behaviors such as excessive sedentary video game playing and television watching in combination with the consumption of energy dense foods such as fruits and vegetables could improve the probability of healthy and sustainable weight loss. These health behaviors may track to adulthood where they may further have an impact of morbidity and mortality [13–15]. Examining these relationships concurrently will provide information whether there is a discordance between intent and engagement in specific behaviors and may provide important information to help derive effective and sustainable pediatric obesity treatment programming.

Data from the National Youth Risk Behavior Survey (YRBS) provides information using a representative sample of US adolescents that can be used to examine relationships between several sedentary behavior, physical activity, and dietary-related variables with weight loss intent [16]. The National YRBS also collects data regarding current perceived weight status, which may be an effect modifier in the aforementioned health behavior associations with weight loss intent. Previous research conducted by Fan and Jin [17] used propensity score matching to examine the relationship between perceived overweight status and weight loss intent and between perceived overweight status and various energy balance related outcomes using pooled YRBS data. The authors observed that adolescents who perceived themselves as overweight had a stronger intention to lose weight but that this perception did not transfer to better eating and exercise habits [17]. Although a number of potential confounding variables were controlled for in their analyses employing propensity score matching, the independent relationships between specific energy balance health behaviors and weight loss intent were not explored nor was the potential moderating effect of perceived weight status [17]. The current study aims to address these gaps using recent 2017 YRBS data. Therefore, the purpose of this study was to examine the associations between specific sedentary behaviors, physical activity-related behaviors, and dietary behaviors with the intent to lose weight in a sample of adolescents from the 2017 US National YRBS. A secondary purpose was to examine these associations using a 2017 YRBS subpopulation of adolescents who self-described themselves as being overweight to test for modifying effects.

## Methods

### Participants

Data were collected in 2017 and analyzed in the spring of 2019. A multi-stage cluster sampling procedure was used to obtain a representative sample of US adolescents. The target population consisted of public and private high schoolers from grades 9 through 12. A weighting factor was applied to each adolescent to adjust for nonresponse and the oversampling of Black and Hispanic adolescents. The final weights were scaled so that the weighted count of students was equal to the total sample size, and the weighted proportions of students in each grade matched population projections [18]. The National YRBS has been approved by CDC’s Institutional Review Board.

All regular public and private school students in the 50 States and the District of Columbia were included in the sampling frame. Schools were systematically selected with probability proportional to enrollment using a random start. One hundred and ninety-two schools were sampled and 144 of the 192 sampled schools participated (75% response rate). All classes in a required subject or all classes meeting during a particular period of the day were included in the sampling frame. Systematic equal probability sampling with a random start was used to select classes from each school. The number of sampled adolescents was 18,324 with 14,765 of the 18,324 sampled students submitting questionnaires with usable data (81% response rate). The overall response rate (school response rate × student response rate) was 60% [16].

### Youth risk behavior survey procedures

YRBS questionnaires were self-administered and adolescents recorded responses on a computer-scannable questionnaire booklet or answer sheet. Local procedures for obtaining parental permission were followed before administering YRBS at any school. Trained data collectors traveled to each participating school to administer the questionnaires. The data collectors read a standardized script to participating students explaining the YRBS to students. All procedures for the YRBS were designed to protect privacy, allowing anonymous and voluntary participation. Students completed the self-administered questionnaire during one class period and recorded responses directly on the computer-scannable booklet or answer sheet. When possible, the students’ desks were spread throughout the classroom. Students who were absent during the YRBS had the option to complete the questionnaire at a later date. Most questions on the YRBS have shown to have a high degree of test-retest reliability with kappa = 61–100% [19].

### Data processing

All variables were dichotomized and most were dichotomized in accordance to the methods used on the 2017 YRBS. The outcome variable was the response item Q69 on the National YRBS that asked “Which of the following are you trying to do about your weight?” The response for item Q69 was numerically recoded 1 = lose weight and 0 = not lose weight. Three sets of predictor variables were analyzed consisting of variables relating to sedentary behavior, physical activity, and diet. Two sedentary behavior variables consisted of response items that asked about television watching (Q80: recoded 1 = 3 or more hours/day, 0 = 2 or less hours/day) and video game playing (Q81: recoded 1 = 3 or more hours/day, 0 = 2 or less hours/day). Three activity-related variables consisted of items asking about daily physical activity (Q79: 1 = 60 min/day, 0 = less than 60 min/day), sports team participation (Q83: 1 = 1 or more sports team in past 12 months, 0 = no sports teams in past 12 months), and engagement in muscular strength exercises (Q95: 1 = 3 or more days/week, 0 = 2 or fewer days/week). The physical activity item (Q79) was alternatively dichotomized in accordance to the US physical activity recommendations for children and adolescents (i.e., 60 min of physical activity every day of the week). Finally, the dietary-related variables consisted of response items that asked about breakfast consumption (Q78: 1 = everyday consumption, 0 = not everyday consumption), fruit consumption (Q71: 1 = 1 or more times in past week, 0 = no consumption in past week), green salad consumption (Q72: 1 = 1 or more times in past week, 0 = no consumption in past week), potato consumption (Q73: 1 = 1 or more times in past week, 0 = no consumption in past week), carrot consumption (Q74: 1 = 1 or more times in past week, 0 = no consumption in past week), and other vegetable consumption (Q75: 1 = 1 or more times in past week, 0 = no consumption in past week). All dietary questions were used to determine healthy food intake.

### Statistical analysis

A probability weight based on sex, race/ethnicity, and grade level was applied to each adolescent to adjust for school and student nonresponse and the oversampling of Black and Hispanic students. The complex survey design, including assigned stratum and primary sampling unit, was accounted for using STATA’s “svyset” prefix command. Of the adolescents who had valid outcome variable scores (Q69), there was less than 10% missing data for every predictor, therefore missing data were not imputed. Weighted analyses used the Taylor Series Linearization variance estimation. For all descriptive characteristics, unweighted and weighted prevalence statistics were reported. Additionally, raw counts and unweighted and weighted prevalence were also reported for the outcome and predictor variables. To examine the associations between sedentary behaviors, physical activity, and specific dietary behaviors with adolescent weight loss intent, weighted logistic regression models were employed. Predictors were entered into the models using block-wise entry. Model 1 consisted of all primary predictor variables and Model 2 consisted of the Model 1 and the potential confounding of age, sex, BMI percentile, and race/ethnicity. BMI percentile, based on CDC growth charts, was used to provide an interpretable BMI metric accounting for adolescent growth and development. Social-economic status was not controlled for because it was not collected on the 2017 National YRBS. Referent levels for all primary predictor variables were categories coded as 0 (see Data Processing). The referent for sex was females and the referent for race/ethnicity was White because this category had the highest prevalence. Subpopulation analyses were also conducted to determine if adolescents who described themselves as overweight (Q88: 1 = slightly or very overweight, 0 = not overweight), significantly modified the observed associations. Subpopulation analyses were carried out using STATA’s “subpop” command. Communication of the results consisted of reporting the unadjusted and adjusted odds ratios (ORs) with corresponding 95% Confidence Intervals. All analyses had an alpha level of *p* < 0.05 and were carried out using STATA v15.0 statistical software package (College Station, Texas, USA).

## Results

Unweighted and weighted prevalence for all descriptive characteristics are reported in Table 1 and the unweighted and weighted prevalence for the outcome and predictor variables are reported in Table 2. A little less than one-half (47.3%) of the sample self-reported a current intention to lose weight and a little less than one-third of the sample self-described themselves as being either a little or very overweight (31.3%). Results from the weighted logistic regression models using the total sample are reported in Table 3. After adjusting for age, sex, BMI percentile, and race/ethnicity, the energy balance-related behaviors that associated with higher odds of weight loss intent included playing video games for 3 or more hours per day (*p* = 0.028) and regular salad consumption (*p* = 0.001). Health behaviors associating with lower odds of weight loss intent included meeting daily physical activity guidelines (*p* < 0.001) and daily breakfast consumption (*p* < 0.001). Being male significantly related to lower odds of weight loss intent (*p* < 0.001) and higher BMI percentile related to higher odds of weight loss intent (*p* < 0.001). Regarding the race/ethnicity covariate, compared to Whites, being Asian (*p* = 0.003) or single Hispanic/Latino (*p* = 0.001) or multiple Hispanic Latino (*p* = 0.028) associated with higher odds of weight loss intent while being Black associated with lower odds of weight loss intent (*p* < 0.001).

**Table 1:** Unweighted and weighted prevalence of observed descriptive characteristics

Variable	Level	Unweighted Count	Unweighted Percent	Weighted Percent
Sex	Females	7526	51.4%	50.7%
	Males	7112	48.6%	49.3%
Age	12 years old or younger	59	0.4%	0.3%
	13 years old	22	0.2%	0.1%
	14 years old	1922	13.1%	11.7%
	15 years old	3586	24.4%	24.5%
	16 years old	3688	25.1%	25.4%
	17 years old	3611	24.6%	24.2%
	18 years old	1796	12.2%	13.4%
Race/Ethnicity	White	6261	44.4%	53.5%
	Asian	648	4.5%	3.5%
	Black/African American	2796	19.4%	13.4%
	Pacific Islander	116	0.8%	0.8%
	American Indian/Alaskan Native	137	1.0%	0.5%
	Hispanic/Latino	1543	10.7%	9.8%
	Multiple-Hispanic/Latino	2104	14.6%	13.1%
	Multiple-Non-Hispanic/Latino	823	5.7%	5.5%

A weighting factor was applied to each adolescent to adjust for nonresponse and the oversampling of Black and Hispanic adolescents

**Table 2:** Unweighted and weighted prevalence of observed predictor and outcome variables

Variable	Level	Unweighted Count	Unweighted Percent	Weighted Percent
Weight Loss Intent	Not intending to lose weight	6079	52.7%	52.9%
	Intending to lose weight	5462	47.3%	47.1%
Weight Self-Perception	Not perceived as overweight	9605	68.7%	68.5%
	Perceived as overweight	4381	31.3%	31.5%
Television Watching	Less than 3 h of television/day	10,790	77.8%	79.3%
	3 or more hours of television/day	3077	22.2%	20.7%
Video Games	Less than 3 h of video game playing/day	7855	56.8%	57.0%
	3 or more hours of video game playing/day	5984	43.2%	43.0%
Physical Activity (PA)	Less than 60 min of PA/day	10,796	75.8%	73.9%
	60 min of PA/day	3442	24.2%	26.1%
Sports Participation	No sports teams	5307	45.3%	45.7%
	At least 1 sports team	6413	54.7%	54.3%
Muscular Strength Exercising	Less than 3 days of strength exercising	5389	50.3%	48.9%
	3 or more days of strength exercising	5324	39.7%	51.1%
Breakfast Consumption	Do not consume breakfast everyday	7783	65.3%	64.7%
	Consume breakfast everyday	4135	34.7%	35.3%
Consume Fruit	Do not consume fruit	1758	12.2%	11.1%
	Consume fruit at least once per week	12,631	87.8%	88.9%
Consume Salad	Do not consume salad	5972	42.6%	40.7%
	Consume salad at least once per week	8053	57.4%	59.3%
Consume Potatoes	Do not consume potatoes	5155	36.8%	34.6%
	Consume potatoes at least once per week	8872	63.2%	65.4%
Consume Carrots	Do not consume carrots	7890	56.4%	54.7%
	Consume carrots at least once per week	6099	43.6%	45.3%
Consume Other Vegetables	Do not consume other vegetables	2720	19.5%	18.5%
	Consume other vegetables at least once per week	11,215	80.5%	81.5%

PA stands for physical activity; a weighting factor was applied to each adolescent to adjust for nonresponse and the oversampling of Black and Hispanic adolescents

**Table 3:** Parameter estimates from the weighted multiple logistic regression model using the total sample

	Model 1		Model 2	
	OR	95% CI	OR	95% CI
Sedentary Behaviors				
3 or More Hours of Television/Day	1.07	0.97–1.20	1.03	0.92–1.18
3 or More Hours of Video Game Playing/Day	1.07	0.95–1.21	**1.15** ^**†**^	1.01–1.31
Activity-Related Behaviors				
60 min of PA/Day	**0.57** ^**†**^	0.51–0.63	**0.66** ^**†**^	0.59–0.73
At least 1 Sports Team	**0.84** ^**†**^	0.76–0.94	0.88	0.76–1.00
3 or More Days of Strength Exercising	0.90	0.80–1.01	1.03	0.92–1.16
Dietary Behaviors				
Consume Breakfast Everyday	**0.61** ^**†**^	0.55–0.69	**0.76** ^**†**^	0.67–0.87
Consume Fruit	**1.47** ^**†**^	1.22–1.77	1.26	0.99–1.61
Consume Salad	**1.43** ^**†**^	1.30–1.58	**1.30** ^**†**^	1.12–1.52
Consume Potatoes	0.90	0.83–1.04	0.90	0.80–1.00
Consume Carrots	0.93	0.82–1.04	0.93	0.82–1.07
Consume Other Vegetables	**1.14** ^**†**^	1.02–1.25	0.99	0.84–1.17
Age (years)			0.99	0.96–1.03
Male			**0.26** ^**†**^	0.23–0.31
BMI %			**1.05** ^**†**^	1.04–1.05
American Indian/Alaskan Native			0.75	0.45–1.25
Asian			**1.70** ^**†**^	1.20–2.41
Black/African American			**0.56** ^**†**^	0.45–0.70
Pacific Islander			0.66	0.34–1.28
Hispanic/Latino			**1.45** ^**†**^	1.17–1.79
Multiple-Hispanic/Latino			**1.21** ^**†**^	1.02–1.45
Multiple-Non-Hispanic/Latino			1.12	0.89–1.42

Outcome is intending to lose body weight; OR stands from Odds Ratio; 95% CI stands for 95% Confidence Interval; PA is physical activity; BMI% is Body Mass Index age and sex percentile; referent for sex is female; referent for race/ethnicity is White; Model 1 = sedentary, physical activity, and dietary-related predictors; Model 2 = Model 1 + potential confounding, *p* < 0.05

bold and † indicates statistical significance, *p* < 0.05

Results from the weighted logistic regression model using a subpopulation of adolescents who consider themselves either a little or very overweight are reported in Table 4. The observed associations using the self-perceived overweight sample included the association between meeting daily physical activity guidelines and lower odds of weight loss intent (*p* < 0.001). However, in contrast to the total sample, daily breakfast consumption (*p* = 0.564) and regular green salad consumption (*p* = 0.559) did not significantly associate with weight loss intent in self-perceived overweight adolescents. Additionally, unlike using the total sample, muscular strength exercising at least 3 days per week significantly associated with higher odds of weight loss intent in self-perceived overweight adolescents (*p* = 0.022) and there was also an association between regular fruit consumption and higher odds of weight loss intent (*p* = 0.012).

**Table 4:** Parameter estimates from the weighted multiple logistic regression model using a subpopulation of adolescents who described themselves as either slightly or very overweight

	Model 1		Model 2	
	OR	95% CI	OR	95% CI
Sedentary Behaviors				
3 or More Hours of Television/Day	0.87	0.67–1.12	0.97	0.72–1.30
3 or More Hours of Video Game Playing/Day	0.85	0.67–1.05	0.93	0.74–1.18
Activity-Related Behaviors				
60 min of PA/Day	**0.57** ^**†**^	0.38–0.81	**0.65** ^**†**^	0.45–0.94
At least 1 Sports Team	0.92	0.70–1.21	0.99	0.73–1.32
3 or More Days of Strength Exercising	**1.31** ^**†**^	1.02–1.69	**1.45** ^**†**^	1.06–2.00
Dietary Behaviors				
Consume Breakfast Everyday	0.86	0.64–1.09	0.92	0.70–1.22
Consume Fruit	**1.89** ^**†**^	1.33–2.70	**1.60** ^**†**^	1.12–2.37
Consume Salad	1.19	0.89–1.59	1.11	0.80–1.54
Consume Potatoes	**0.71** ^**†**^	0.53–0.97	0.73	0.52–1.01
Consume Carrots	1.10	0.83–1.46	1.19	0.87–1.62
Consume Other Vegetables	1.34	0.88–2.03	1.13	0.71–1.76
Age (years)			0.93	0.83–1.05
Male			**0.44** ^**†**^	0.32–0.61
BMI %			1.00	0.99–1.01
American Indian/Alaskan Native			0.76	0.22–2.68
Asian			1.28	0.64–2.59
Black/African American			1.18	0.74–1.89
Pacific Islander			3.77	0.55–26.0
Hispanic/Latino			**2.13** ^**†**^	1.17–3.89
Multiple-Hispanic/Latino			**1.62** ^**†**^	1.03–2.51
Multiple-Non-Hispanic/Latino			1.34	0.69–2.62

Outcome is intending to lose body weight; OR stands from Odds Ratio; 95% CI stands for 95% Confidence Interval; PA is physical activity; BMI% is Body Mass Index age and sex percentile; referent for sex is female; referent for race/ethnicity is White; Model 1 = sedentary, physical activity, and dietary-related predictors; Model 2 = Model 1 + potential confounding, *p* < 0.05

bold and † indicates statistical significance, *p* < 0.05

## Discussion

The purpose of this study was to examine the associations between specific sedentary behavior, physical activity, and dietary-related variables with adolescent weight loss intent using information collected on the 2017 US National YRBS. The results indicated that meeting daily physical activity and daily breakfast consumption associated with lower odds of weight loss intent and that playing video games for 3 or more hours per day and regular salad consumption associated with higher odds of weight loss intent. Adolescents who perceived themselves as overweight slightly modified these results as only meeting daily physical activity guidelines associated with lower odds of weight loss intent and muscular strength training and fruit consumption associated with higher odds of weight loss intent. Although several significant associations were observed, no causal relationships can be established because of the cross-sectional design. A discussion of these findings and implications for public health practice are discussed further.

The salient findings were that specific energy balance-related behaviors significantly associated with weight loss intent. Specifically, meeting daily physical activity guidelines and daily breakfast consumption associated with lower odds of weight loss intent and playing video games for 3 or more hours per day and regular salad consumption associated with higher odds of weight loss intent. The findings suggest that favorable associations between the engagement in health behaviors and weight loss intent were mixed. The associations between physical activity and breakfast consumption with lower odds of weight loss intent and the association between video game playing and higher odds of weight loss intent may compromise healthy and sustainable weight loss while the association between salad consumption and higher odds of weight loss intent may facilitate healthy weight loss efforts. Therefore, there is a discordance between weight loss intent and the engagement in some energy-balance-related health behaviors. The recommendation for US children and adolescents for physical activity is 60 min of physical activity every day [20]. It has been shown that the addition of physical activity to diet programs may promote more sustainable weight loss compared to diet alone [21–23]. Although diet by itself may yield significant weight loss, especially in the morbidly obese, lower levels of sedentary behavior and higher levels of physical activity may supplement and may help sustain positive effects [24]. This, in addition to the other positive benefits of low sedentary behavior and higher habitual physical activity such as improved cognitive functioning, improved emotional wellbeing, improved health-related fitness, and a decrease in non-communicable disease risk needs to be communicated effectively to adolescents who intend to lose weight [25–27].

The findings also revealed that daily breakfast consumption associated with lower odds of weight loss intent. The influence of breakfast consumption on daily energy intake and overall health is debatable [28]. Refraining from consuming breakfast may yield an overall negative daily energy balance, which may lead to weight loss [29, 30]. However, longitudinal observational studies have found that women who consume breakfast everyday were less likely to become obese those women who did not [31] and eating breakfast at home has been associated with the prevention of weight regaining after dieting [32]. Furthermore, consuming breakfast has also been correlated with higher levels of cognitive functioning and academic achievement in adolescents [33, 34]. Therefore, not eating breakfast everyday may not be a favorable health behavior in terms of cognitive functioning or for performance in academic classes but yet may yield negative energy balances for weight loss. Youth seeking to lose weight should be properly educated on the benefits and limitations of breakfast consumption and how consuming breakfast may facilitate consumption of a healthy diet [35].

Results of the secondary analysis revealed that in a subpopulation of adolescents who describe themselves as overweight, there was some modified associations compared to those observed using the total 2017 YRBS sample. The lone health behavior predictor associated with lower odds of weight loss intent in the subpopulation sample was meeting daily physical activity guidelines. However, unlike the total sample, the muscular strength exercising at least 3 days per week associated with higher odds of weight loss intent. It has been shown that youth with obesity tend to have impaired muscular fitness compared to normal weight peers [36]. Therefore, the relationship between muscular strength exercising and weight loss intent in perceived overweight adolescents is an encouraging finding [37]. Muscular strength training may improve motor skills, lower injury rates, and improve self-esteem and interest in fitness [38]. However, its direct role in weight loss is not as established but may still improve certain health and psychosocial parameters in obese youth [39, 40]. Interventions have shown that using a combination of both aerobic and resistance (muscular strength) training yielded greater reductions in body weight and greater improvements in body fat and cardiorespiratory fitness compared to control and resistance training only groups [41]. A message to incorporate aerobic exercise to manifest negative energy balance is also needed however, despite participation in muscular strength training programs. The only other predictor associating with higher odds of weight loss intent in the subpopulation analysis was regular fruit consumption. Although this is a positive finding given the health benefits of fruit consumption, there were no other favorable associations found between regular vegetable consumption with weight loss intent. The literature yields mixed findings whether fruit or vegetable consumption alone or in combination yields weight loss or attenuated weight gain in children independent of other energy balance-related health behaviors, yet encouraging regular consumption of fruit and vegetables in adolescents is still recommended for overall health [42].

The results of this study show a discordance in the associations between weight loss intent and the engagement in specific energy balance-related health behaviors. Using 2010 YRBS data, Kakinami et al. [43] also found that weight loss attempts alone do not affect the likelihood of meeting recommendations for diet and physical activity. This potentially suggests that adolescents who intend to lose weight do not know how or what specific health behaviors to engage in to yield a negative energy balance, particularly with regards to sedentary behavior and physical activity. The specific relationships that support this conjecture in the current study was the association between video game playing for 3 or more hours per day and higher odds of weight loss intent and the association between meeting daily physical activity guidelines and higher odds of weight loss intent. Although diet has been shown to be a major factor in creating a negative energy balance, physical activity has at least a supporting role and provides numerous other benefits independent of dietary behaviors [44]. Current recommendations are that both diet and physical activity need to be engaged in to yield efficient and sustainable negative energy balance that leads to weight loss [45, 46]. The role of physical activity may be especially important during adolescence as a longitudinal study found that resting energy expenditure dramatically decreases during puberty [47]. These messages could be more effectively communicated to youth trying to lose weight as meeting daily physical activity guidelines and reducing behaviors such as excessive sedentary video game playing in combination with the consumption of energy dense foods such as fruits and vegetables could improve the probability of healthy and sustainable weight loss.

Strengths of this study include the use of a large and national representative sample of US adolescents and the use of multiple sedentary behavior, physical activity, and dietary variables to associate with weight loss intent. There are also limitations to this study that must be considered. First, the study design was cross-sectional, therefore no causal inferences can be made; follow-up studies need to employ longitudinal research designs. Along these lines, the direction of association was most likely intent leading to engagement in health behaviors, however the study design also precludes examination of the directionality of association. Future research should employ longitudinal research designs using weight loss intent as the predictor and specific energy-balance-related health behaviors as outcomes. Second, all variables were collected using self-report, therefore there is potential for recall and social desirability bias. Third, only one subpopulation of the National YRBS was examined to test for effect modification (overweight perception), future research should examine the relationships with other variables which may contribute to effect modification. Finally, psychosocial mediators such as perceived social support and self-efficacy may play a role in the engagement in health behaviors. These variables were not collected and may have confounded the association between health behaviors and weight loss intent.

## Conclusion

In conclusion, meeting daily physical activity guidelines and daily breakfast consumption associated with lower odds of weight loss intent and 3 or more hours of video game playing and regular salad consumption associated with higher odds of weight loss intent in a representative sample of adolescents from the 2017 US National YRBS. Adolescents who perceived themselves as overweight slightly modified these results. These results indicate that there is a gap between intent to lose weight and engagement in specific energy balance-related health behaviors. This is especially true for physical activity as the association between meeting daily physical activity guidelines and lower odds with weight loss intent was robust and also held within the perceived overweight subpopulation. It appears that adolescents who regularly consume salads associate with higher odds of weight loss intent however regular breakfast consumption related to lower odds of weight loss intent. Although consumption of breakfast contributes to positive energy balance, skipping breakfast has been shown to associate with a higher risk of weight regain and may also compromise cognitive functioning. These results indicate that adolescents who intend to lose weight should be aware of current health behaviors and how engagement in these health behaviors contribute to energy balance and to overall health.

## Data Availability

The datasets analyzed during the current study are available in the CDC National YRBS website, https://www.cdc.gov/healthyyouth/data/yrbs/data.htm.

## Abbreviations

BMI: Body Mass Index
CDC: Centers for Disease Control and Prevention
CI: Confidence Interval
OR: Odds Ratio
YRBS: Youth Risk Behavior Survey
